# Impact of Cell-surface Antigen Expression on Target Engagement and Function of an Epidermal Growth Factor Receptor × c-MET Bispecific Antibody*

**DOI:** 10.1074/jbc.M115.651653

**Published:** 2015-08-10

**Authors:** Stephen W. Jarantow, Barbara S. Bushey, Jose R. Pardinas, Ken Boakye, Eilyn R. Lacy, Renouard Sanders, Manuel A. Sepulveda, Sheri L. Moores, Mark L. Chiu

**Affiliations:** From ‡ Janssen Research and Development, LLC, Spring House, Pennsylvania 19477 and; § Janssen Diagnostics, Janssen Research and Development, Huntingdon Valley, Pennsylvania 19104

**Keywords:** cancer therapy, cell-surface receptor, epidermal growth factor (EGF), epidermal growth factor receptor (EGFR), hepatocyte growth factor/scatter factor (HGF/SF), bispecific antibody, c-MET, quantitative flow cytometry

## Abstract

The efficacy of engaging multiple drug targets using bispecific antibodies (BsAbs) is affected by the relative cell-surface protein levels of the respective targets. In this work, the receptor density values were correlated to the *in vitro* activity of a BsAb (JNJ-61186372) targeting epidermal growth factor receptor (EGFR) and hepatocyte growth factor receptor (c-MET). Simultaneous binding of the BsAb to both receptors was confirmed *in vitro*. By using controlled Fab-arm exchange, a set of BsAbs targeting EGFR and c-MET was generated to establish an accurate receptor quantitation of a panel of lung and gastric cancer cell lines expressing heterogeneous levels of EGFR and c-MET. EGFR and c-MET receptor density levels were correlated to the respective gene expression levels as well as to the respective receptor phosphorylation inhibition values. We observed a bias in BsAb binding toward the more highly expressed of the two receptors, EGFR or c-MET, which resulted in the enhanced *in vitro* potency of JNJ-61186372 against the less highly expressed target. On the basis of these observations, we propose an avidity model of how JNJ-61186372 engages EGFR and c-MET with potentially broad implications for bispecific drug efficacy and design.

## Teaser

**Background:** Cancer cells express surface antigens at different levels from normal cells.

**Results:** Differences in EGFR and c-MET receptor density levels influenced the *in vitro* activity of an EGFR × c-MET bispecific antibody.

**Conclusion:** Consideration of target expression levels is important for bispecific design.

**Significance:** In addition to multiple pathway targeting, the unique avidity of bispecific antibodies contributes to their promise for cancer therapy.

## Introduction

The modulation of a signaling pathway by a therapeutic monoclonal antibody (mAb) requires reaching a threshold of target engagement to achieve efficacy. This threshold is partially determined by the affinity and avidity of the mAb for its target (1, 2). Thus, target antigen density can play a prominent role in guiding mAb behavior (2). *In vitro* models where antigen density can be systematically controlled and dependent biological responses evaluated have been reported (3). However, they are difficult to generate in therapeutically relevant cellular backgrounds that are based on tumor models. It has been shown that differences in antibody-dependent cellular cytotoxicity measured against certain cancer targets (*e.g.* CD20) suggest the existence of a minimum expression level below which activity cannot be achieved (4–7). Beers *et al.* (8) demonstrated that *in vitro* effector functions such as antibody-dependent cellular cytotoxicity, antibody-dependent cellular phagocytosis, and complement-dependent cytotoxicity can be modified by treatment-induced changes in target expression levels.

Malignant cells frequently have altered cell-surface protein expression compared with normal cells (9–11). These expression differences have broad implications for target selection, tissue penetration, drug specificity, and efficacy, and in a clinical setting they have broad implications for the effects of diagnosis, disease monitoring, and treatment modulation (1, 11–13). A number of recent reviews highlight the potential importance of intratumoral heterogeneity, likely intrinsic to many cancer types, as a source of resistance to currently available therapies (14–16). In the case of non-small cell lung cancers (NSCLC),^2^ mutational heterogeneity in epidermal growth factor (EGF) receptor (EGFR) provides one possible escape mechanism for patients exhibiting resistance to tyrosine kinase inhibitors (14, 15, 17, 18). Activation of the hepatocyte growth factor receptor (c-MET, mesenchymal endothelial transition) pathway, which like EGFR can drive cellular proliferation, provides another major route of resistance (19, 20). The simultaneous targeting of both signaling pathways with an EGFR x c-MET bispecific antibody (BsAb) could produce synergies that more effectively block tumor proliferation and metastasis (21–23).

An assessment of the therapeutic value of a BsAb entails a comparison of binding and functional activity of the BsAb with that of the individual parental mAbs that comprise it. The role that the surface density of each target plays in determining the efficacy of a BsAb, particularly in the context of heterogeneous cancer cell populations, remains to be thoroughly explored. We are interested in how JNJ-61186372 engages EGFR and c-MET on the cell surface and how the relative expression of its two targets influences its behavior. Starting with well established methods for receptor quantitation using flow cytometry, we have applied an improved set of tools made available by controlled Fab-arm exchange (cFAE) technology (24) to explore whether expression data correlate to receptor density and whether *in vitro* differences in activity might arise from differences in the relative expression of EGFR and c-MET.

We determined the cell-surface density of EGFR and c-MET in a panel of cancer cell lines grown under uniform conditions using flow cytometry. The application of flow cytometry methods to the quantitation of cell-surface antigens has become widespread since its introduction in the 1980s (13, 25). The term quantitative flow cytometry (QFCM) was coined to describe a set of methodologies for quantitation designed to standardize procedures and reagents to minimize inter-laboratory variability (11). In contrast to older qualitative methods, the assignment of defined values to describe antigen densities has demonstrated value in a wide range of applications (26). Notable studies using QFCM include the evaluation of differences in lymphocyte antigen expression in HIV (27–29), the characterization of malignancy, and the identification of prognostic indicators in leukemia (27, 30–32). QFCM has also been applied to the determination of genetic heterozygosity, the diagnosis of sepsis, and the study of multidrug resistance (11, 34). Nonetheless, the inherent challenges associated with QFCM have left standardization a lingering challenge for the scientific community (25, 35).

Although determining the number of epitopes per cell for a given antigen is typically the goal of QFCM, quantitative data are routinely generated with monoclonal Abs conjugated to a fluorochrome and are reported as the antibody-binding capacity (ABC) (36). However, the bivalency of monoclonal Abs obscures the precise determination of epitope number. Although investigation of the valency of Ab binding could potentially improve the accuracy of density measurements, these studies are typically not performed due to their laborious nature (37, 38). The advent of cFAE technology to generate bispecific Abs creates a novel and convenient opportunity for quantifying surface antigens. The combination of one antigen-specific Fab arm, and a binding arm that binds to an inert antigen that is not present on the target cell, enables researchers to construct full-length monovalent Abs. The use of monovalent Abs for quantitation removes inaccuracies introduced by the valency of binding when using conventional monoclonal Abs and removes the need to design and prepare constructs of different sizes (*e.g.* Fab and scFv) to achieve monovalency. Using different architectures of binders can affect the nature of interactions and thereby make systematic comparisons difficult to achieve. Because monovalent Abs lack avidity, the use of high affinity antigen-specific variants is essential (39). Equally important to our method is the application of labeling and purification methodologies that result in a 1:1 fluorochrome to protein (F/P) molar labeling ratio. The latter obviates the need to account for multiple and/or heterogeneous labeling ratios when interpreting fluorescent data. Although not the primary focus of this investigation, the ability to compare a monovalent full-length Ab to its bivalent parental Ab also makes possible a thorough investigation of the stoichiometry of binding.

In this paper, we demonstrate how BsAbs against EGFR and c-MET generated using cFAE can be used for accurate receptor quantitation, as well as to gain insight into the mechanisms of bispecific target engagement and their biological consequences in a cellular context. An approach to characterization of dual target engagement is also presented for an array of lung and gastric cancer cell lines that reflect diversity in the mutational status of EGFR and c-MET (Table 1). We also demonstrate the simultaneous binding of JNJ-61186372 to both the EGFR and c-MET via SPR and cell-based assays.

**TABLE 1:** **Genotypes of EGFR and c-MET in cancer cell lines** The following abbreviations were used: ACA, adenocarcinoma; del, deletion mutant; BAC, bronchioalveoloar carcinoma; MEC, mucoepidermoid carcinoma; ND, not determined; SCC, squamous cell carcinoma; UNK, unknown, WT, wild type; Y, yes; N, no.

Cell line	Origin	EGFR		c-MET	
		Genotype	Amplified (copies/cell)	Genotype	Amplified (copies/cell)
H292	Lung MEC	WT	N	WT	N
SKMES-1	Lung SCC	WT	N	WT	N
HCC827	Lung ACA	del (E746, A750)	Y (19)	WT	N
H1975	Lung ACA	L858R, T790M	N	WT	N
H3255	Lung ACA	L858R	Y (12)	WT	N
H1650	Lung BAC	del (E746, A750)	Y (4)	WT	N
HCC4006	Lung ACA	del (L747, S752)	Y (5)	WT	N
HCC2935	Lung ACA	del (E746, A750)	N	WT	N
H820*^a^*	Lung ACA	del (E746, A750),	UNK*^a^*	WT	Y (4–6)
		T790M			
H1993*^a^*	Lung ACA	WT	UNK*^a^*	WT	Y (UNK*^a^*)
SNU-5	Stomach ACA	WT	N	WT	Y (9)

*^a^* Not in Cancer Cell Line Encyclopedia.

## Experimental Procedures

### 

#### 

##### Cell Culture

The 11 tumor cell lines included H292, SKMES-1, HCC827, H1975, H3255, H1650, HCC4006, HCC2935, H820, H1993, and SNU-5. The tumor cell lines were obtained from the American Type Culture Collection (ATCC, Manassas, VA). They were cultured in 150-cm^2^ tissue culture flasks under standard culture conditions (37 °C, 5% CO_2_, 95% humidity) using ATCC-recommended media formulations. H292, HCC827, H1975, H3255, H1650, HCC4006, HCC2935, H820, and H1993 were grown in RPMI 1640 medium +GlutaMAX^TM^ + 25 mm HEPES (Life Technologies, Inc.), 10% heat-inactivated fetal bovine serum (Life Technologies, Inc.), 0.1 mm nonessential amino acids (NEAA, Life Technologies, Inc.), and 1 mm sodium pyruvate (Life Technologies, Inc.). SKMES-1 cells were cultured in minimum essential medium (MEM) + GlutaMAX^TM^ without HEPES (Life Technologies, Inc.), 0.1 mm NEAA (Life Technologies, Inc.), and 1 mm sodium pyruvate (Life Technologies, Inc.). SNU-5 was cultured in Iscove's modified Dulbecco's medium + l-glutamine + 25 mm HEPES (Life Technologies, Inc.), 20% heat-inactivated fetal bovine serum (Life Technologies, Inc.), 0.1 mm NEAA (Life Technologies, Inc.), and 1 mm sodium pyruvate (Life Technologies, Inc.). Media were routinely changed two to three times weekly. Subconfluent cell monolayers were passaged using Accutase (Sigma). Cells were harvested for receptor quantitation at ∼80% confluence, and passage number was never allowed to exceed 15 passages. To dissociate cells for receptor quantitation, enzyme-free Cellstripper (Corning) was used to avoid receptor proteolysis. The minimum required incubation time at 37 °C for cell detachment was optimized for each cell type. All cell lines were shown to be sterile and certified mycoplasma-free. EGFR and c-MET gene amplification status and copy number were obtained from the Broad-Novartis Cancer Cell Line Encyclopedia.

##### Ab Expression and Purification

Separate plasmids encoding Ab heavy chains and light chains were co-transfected at a 3:1 (light chain/heavy chain) molar ratio into Expi293F cells following the transfection kit instructions (Life Technologies, Inc.). Cells were spun down 5 days pos-transfection, and the supernatant was passed through a 0.2-μm filter inside a laminar flow cabinet. The amount of IgG was quantified using Octet (ForteBio). The NaCl concentration was adjusted to 0.5 m, and the supernatant was filtered again. Purification was carried out using prepacked 1 or 5 ml of HiTrap Mabselect SuRe^TM^ protein A columns (GE Healthcare) on an ÄKTA FPLC instrument (GE Healthcare). Briefly, the column was equilibrated with 10 column volumes (CVs) of Buffer A, PBS, pH 7.4. Supernatant was loaded onto the column at ∼10 mg of protein per ml of resin. The column was washed with 10 CVs of Buffer A to remove unbound material. Protein was eluted with 5 CVs of 100% Buffer B (50 mm citrate, pH 3.5). The eluate was buffer-exchanged into PBS using a HiPrep 26/10 desalting column (GE Healthcare). Peak fractions were pooled and filtered through a low protein-binding 0.2-μm syringe filter (Pall Corp.) inside a laminar flow cabinet. Protein concentration was determined by UV absorbance at 280 and 310 nm. Quality was assessed by high performance size-exclusion chromatography (SEC) and SDS-PAGE of reduced and nonreduced samples.

##### cFAE to Generate BsAbs

Human IgG1 BsAbs were produced from the two purified bivalent parental Abs according to Labrijn *et al.* (24). Briefly, each parental Ab had been modified to carry a single complementary matched mutation in its third constant domain (CH3), K409R and F405L. The two parental Abs, IgG1-F405L and IgG1-K409R, were mixed in equimolar amounts and allowed to undergo recombination at 31 °C for 5 h in the presence of 75 mm of the mild reducing agent 2-mercaptoethylamine-HCl (Sigma). The 2-mercaptoethylamine-HCl was removed by three rounds of PBS dialysis using a Slide-A-Lyzer cassette (Thermo Scientific) as per the manufacturer's instructions. The dialyzed solution was passed through a low protein-binding 0.2-μm syringe filter (Pall Corp.) in a laminar flow cabinet. BsAb quality was assessed by SDS-PAGE, high performance-SEC and cation-exchange liquid chromatography. BsAb yields were typically > 95%.

##### Binding of EGFR and c-MET via SPR

To determine binding of c-MET and EGFR arms of JNJ-61186372, a ProteOn experiment was performed by having recombinant human c-MET-Fc-His (R&D Systems, catalog no. 1095-ER) first captured onto an HTG sensor chip (Bio-Rad catalog no. 176-5031). After the anti-EGFR/c-MET mAbs or JNJ-61186372 was injected, the sensorgrams were fitted using a 1:1 binding model to determine kinetics and affinity. The experiments to determine affinity for EGFR were conducted by first capturing JNJ-61186372 with an anti-Human Fcγ-specific Ab (Jackson ImmunoResearch) covalently immobilized on a GLC sensor chip (Bio-Rad catalog no. 176-5011) using amine coupling chemistry, according to the manufacturer's instructions. Subsequently, different concentrations of analyte, the monomeric extracellular domain (ECD) of EGFR (R&D Systems, catalog no. 1095-ER), were injected over the chip surface. Capture surface regeneration was performed using 10 nm glycine, pH 1.5.

Co-binding of c-MET and EGFR to JNJ-61186372 was analyzed by SPR using the ProteOn XPR instrument. About 140 resonance units of c-MET-Fc chimera bearing a His tag was captured on nickel-activated HTG sensor chip surface. The recombinant human HGF receptor/c-MET-Fc-His capture was followed by co-injection of JNJ-61186372 (injection 1), followed by serially diluted EGFR monomer (injection 2). The co-injection was performed at a flow rate of 50 μl/min for 216 s. The dissociation was monitored for 900 s. Regeneration of the capture surface was performed using 300 mm EDTA, pH 8.5 (Bio-Rad), at a flow rate of 30 μl/min and 800-s contact time. Before the next capture was performed, 60 s of PBS buffer containing Tween 20 (Bio-Rad) was injected at 100 μl/min flow rate. Data were analyzed with ProteOn Manager^TM^ software version 3.1. Capture levels were determined by grouping all sensorgrams by ligands and creating a report point where the average was taken for ∼10 s at the end of the injection for capture. Ligand channel reference was achieved by subtracting the signal from an empty ligand (buffer only) channel. A double correction was then performed using the analyte channel with anti-EGFR or c-MET Abs or the bispecific Ab only. The injection start point for the JNJ-61186372 was re-adjusted to start at zero at ∼200 s into the run time. The data were then fitted using a Langmuir 1:1 interaction model. The kinetic rate constants, *k_a_* and *k_d_*, were derived for each reaction. *K_D_* value was calculated from the *k_d_*/*k_a_* ratio.

##### Conjugation of Abs with (R)-Phycoerythrin (R-PE)

Abs were conjugated to R-PE (ProZyme) using heterobifunctional chemistry. The R-PE was activated using sulfo-sulfosuccinimidyl 4-(*N*-maleimidomethyl)cyclohexane-1-carboxylate (Pierce) for 60 min. Activated R-PE was separated from free sulfo-sulfosuccinimidyl 4-(*N*-maleimidomethyl)cyclohexane-1-carboxylate by gel filtration chromatography. The Abs were reduced using dithiothreitol (DTT, Sigma) for 30 min. The reduced Abs were separated from free DTT by gel filtration chromatography. The activated R-PE was covalently coupled to the reduced Abs for 90 min. The reaction was quenched with *N*-ethylmaleimide (Fluka) for 20 min. The PE-conjugated Abs were purified by size-exclusion chromatography (SEC) using a Tosoh TSKgel G3000SW column in 100 mm sodium phosphate, 100 mm sodium sulfate, 0.05% sodium azide, pH 6.5, on an AKTA explorer FPLC system. The R-PE-conjugated Abs were stored at 2–8 °C in light-occlusive containers. For analytical SEC, the pooled fractions were applied to a Tosoh TSKgel G3000SWxl column in 100 mm sodium phosphate, 100 mm sodium sulfate, 0.05% sodium azide, pH 6.5. The samples were quantified by absorbance at 280 nm normalized for the contribution of conjugated R-PE using Equation 1,

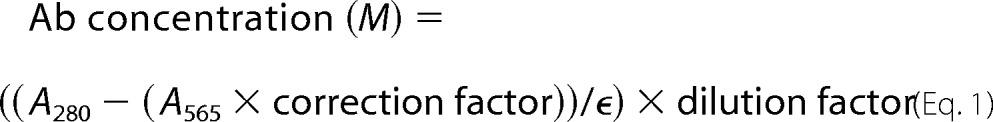
 where *A*_280_ is the absorbance value at 280 nm of the Ab-PE conjugate; *A*_565_ is the absorbance value at 565 nm of the Ab-PE conjugate; the correction factor equals the *A*_280_/*A*_565_ of a PE solution, and ϵ is the extinction coefficient of the Ab at 280 nm. The 1:1 labeling ratio was confirmed using Equation 2,

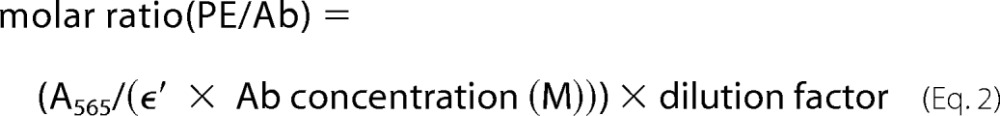
 where *A*_565_ is the absorbance value at 565 nm of the Ab-PE conjugate, and ϵ′ is the molar extinction coefficient value of the fluorescent dye. Following quantitation, samples were diluted to 40 μg/ml working stocks in PBS, pH 7.5, with 0.5% BSA and 0.1% (w/v) NaN_3_, plus 0.01% (w/v) Silwet (Momentive Performance). Confirmation of the free R-PE component was determined using Equation 3,

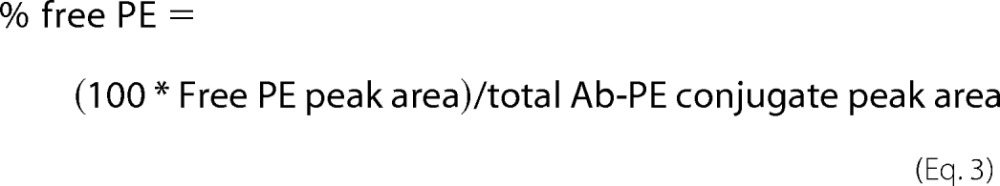
 where the peak areas are determined by analytical SEC.

##### Flow Cytometry Staining

Immediately following dissociation, the cells were assessed for viability by exclusion of 0.4% (w/v) trypan blue (Life Technologies, Inc.). Cell number and viability were calculated using a C-chip hemocytometer (Incyto, Covington, GA). Cells were resuspended at 1 × 10^6^ cells/ml in BSA Stain Buffer (BD Biosciences), from which point cells were rigorously kept at 4 °C to prevent receptor internalization. Fixation of cells was avoided due to the potential effects of paraformaldehyde on Ab dissociation rates and/or epitope integrity (28, 38). Fc receptors (although reportedly not present on these cells) were blocked with 5 μl per test of human TruStain FcX^TM^ (BioLegend, San Diego) for 30 min at 4 °C. Cells (1 × 10^5^ per well) were transferred to 96-well V-bottom microplates (Greiner, Monroe, NC). Cells were incubated on ice for 1 h with serial dilutions of in-house R-PE-labeled c-MET, EGFR, or JNJ-61186372 at empirically determined saturating concentrations. Commercial Abs used included anti-human HGFR/c-MET-PE (R&D Systems, Minneapolis, WI) and anti-human EGFR-PE (BioLegend, San Diego). Isotype-matched controls included mouse IgG1i control-PE (R&D Systems) and mouse IgG2b/*k* isotype control-PE (BioLegend, San Diego). Isotype controls were applied to cells at the highest concentration used for the respective receptor-specific Ab. Cells were washed twice with 150 μl of BSA Stain Buffer and resuspended in 250 μl of BSA Stain Buffer containing 1:50 diluted DRAQ7 live/dead stain (Cell Signaling Technology, Danvers, MA). Single stain controls for PE and DRAQ7 were also included. Samples were read on either a FACSCalibur (BD Biosciences) or MACSQuant flow cytometer (Miltenyi Biotec, Auburn, CA). The data collection channels for PE/DRAQ7 were as follows: FL2/FL4 (FACSCalibur) and B2/B4 (MACSQuant). Live cells were gated on DRAQ7 exclusion, and the geometric mean fluorescence intensity (MFI) was determined for at least 5000 live events collected at a low flow rate. FlowJo vX software (Tree Star Inc.) was used for analysis.

##### Determination of Antigen Density

Receptor density values are reported as the ABC. ABC values were derived from standard curves generated with QuantiBRITE^TM^ PE beads (BD Biosciences). QuantiBRITE^TM^ PE beads consist of four populations of microspheres that are each conjugated to a distinct number of PE molecules per bead, as developed by Iyer *et al.* (28). The beads were run on the same day and at the same photomultiplier tube settings as the test samples. The QuantiBRITE^TM^ PE beads were evaluated as a biological calibrator against CD4 on human T cells using PE-Leu3a (BioLegend) and found to approximate previously reported ABC values (25, 38). The Quantum^TM^ (*R*)-PE MESF (Bangs Laboratories, Inc., Fishers, IN) and Quantum^TM^ Simply Cellular® anti-mouse IgG (Bangs Laboratories, Inc.) were used to empirically confirm the reported 1:1 F/P ratio of the commercial EGFR and c-MET Abs. To calculate ABC values, the geometric means for the four QuantiBRITE^TM^ PE singlet bead populations were derived in FlowJo. Linear regression was used to create a standard curve in GraphPad Prism 6 (GraphPad Software, Inc.) of the log_10_(number of PE molecules/bead) *versus* the log(MFI). *R*^2^ values were typically ≥0.99. ABC values for the PE-Abs were interpolated from the Quanti-BRITE^TM^ PE standard curve. ABC values presented here are the specific antibody binding capacity calculated by subtracting the ABC values from an isotype-matched control from the ABC of the EGFR or c-MET Abs, rounded to three significant figures.

##### Heterodimerization and Homodimerization Enzyme Fragment Complementation Assay

The EGFR homodimerization and EGFR/c-MET heterodimerization assays were carried using PathHunter® enzyme fragment complementation (EFC) technology (40, 41). Briefly, the extracellular domains of c-MET(1–960) and EGFR(1–679) were C-terminally fused to a complementary inactive fragment of β-galactosidase (β-gal) using standard molecular biology techniques. Constructs were transfected into parental U-2 OS (U2OS) cells purchased from ATCC. Stably transfected U-2 OS clones were analyzed for expression and membrane localization by flow cytometry using PE-labeled anti-EGFR and anti-c-MET Abs. For the EFC assays, cells were plated in quadruplicate in 20 μl of assay media on 384-well plates with 5000 cells/well. Test compounds were serially diluted 1:3 in 0.1% bovine serum albumin (BSA)/PBS such that the highest concentration applied was 10 μg/ml. Recombinant human epidermal growth factor (EGF) and hepatocyte growth factor (HGF) stock solutions were diluted in the same manner as the Abs. EGFR homodimer and c-MET homodimer cell lines were also constructed as positive controls for PathHunter technology and to demonstrate specificity. Treatment-induced heterodimerization or homodimerization of recombinant receptors tagged with complementary β-gal fragments resulted in regeneration of fully functional enzyme whose activity can be monitored via chemiluminescence. Incubations were carried out for 6 h at room temperature. PathHunter® flash detection reagent was added to cells, followed by a 60-min incubation at room temperature. Luminescence, reported as relative light units, was recorded on an EnVision Multilabel Reader (PerkinElmer Life Sciences) at 1 count/s. Nonlinear regression analysis was performed in GraphPad Prism 6 (GraphPad Software, Inc.) using a four-parameter logistic (4PL) model with no constraints. For conversions of g/ml to nanomolar, we used the following molecular mass values HGF to be 80 kDa, mAb to be 150 kDa, EGF to be 6.2 kDa.

##### Receptor Phosphorylation Assays

Receptor phosphorylation assays were performed using phospho-Met (Tyr-1349) assay whole cell lysate kit (Meso Scale Discovery) and phospho-EGFR (Tyr-1173) assay whole cell lysate kit (Meso Scale Discovery). All assay reagents were prepared from provided concentrates according to the manufacturer's instructions. On day 1, cells were plated in 96-well tissue culture-treated plates at 8000 cells/well (H1993 and H292) or 20,000 cells/well (SNU-5) in 100 μl of growth medium and incubated overnight at 37 °C, 5% CO_2_. On day 2, overnight serum starvation was initiated with serum-free medium supplemented with 1 mm sodium pyruvate and 0.1 mm NEAA (Life Technologies, Inc.). On day 3, medium was removed, and cells were incubated for 1 h at 37 °C, 5% CO_2_ with Abs in 50 μl of starvation media. Either 50 ng/ml EGF or 100 ng/ml HGF was then added to Ab-treated and control wells; starvation medium was added to vehicle control wells. Cells were incubated for 15 min at 37 °C, 5% CO_2_; treatment was then removed, and cells were lysed in 70 μl/well of lysis buffer. Thirty μl of neat lysates were transferred to pre-coated, pre-blocked MSD 96-well multiarray plates and incubated for 1 h with shaking at room temperature. Plates were washed three times with 1× Tris Wash Buffer (300 μl/well), and 25 μl/well of ice-cold detection Ab solution (SULFO-TAG anti-total EGFR Ab or SULFO-TAG anti-total c-MET Ab) was added, followed by incubation for 1 h with shaking at room temperature. Detection Ab solution was decanted; plates were washed three times, and 150 μl of Read Buffer was added per well. Electrochemiluminescence (ECL) was recorded on a SECTOR® Imager 6000 (Meso Scale Discovery) instrument using standard 96-well detection parameters. Data were plotted as the logarithm of Ab concentration *versus* ECL signal. IC_50_ values were calculated in GraphPad Prism 6 (GraphPad Software, Inc.) using a 4PL model, and data from multiple experiments were used to calculate mean IC_50_ ± S.E., rounded to two significant figures.

##### mRNA Expression Profiling

Expression profiling was performed by GeneWiz, Inc. (South Plainfield, NJ), using a quantitative real time PCR assay. RNA was extracted from cell pellets of NSCLC samples and normal lung cells using the RNeasy Plus mini kit (Qiagen). The concentration, *A*_260_/*A*_280_ UV absorbance ratio, and *A*_260_/*A*_230_ UV absorbance ratio of the RNA samples were measured by NanoDrop (Thermo Scientific). Complementary DNA was generated from each RNA sample using the high capacity cDNA reverse transcription kit (Invitrogen). Quantitative PCR assays, consisting of primers and probes used to detect EGFR, c-MET, and B2M (reference control gene) were purchased from Life Technologies, Inc. Quantitative polymerase chain reactions were assembled in 384-well plates using TaqMan® PCR Master Mix (Life Technologies, Inc.). Each PCR assay was multiplexed with the B2M assay in a 10-μl reaction containing 0.9 μm forward and reverse primers, 0.25 μm each probe, and 4 ng of cDNA. Amplification consisted of single cycles of 2 min at 50 °C and 10 min at 95 °C, followed by 40 cycles of 15 s at 95 °C, 1 min at 60 °C. Reactions were performed on the 7900HT Real Time PCR instrument (Applied Biosystems). Each sample was assayed in four replicate wells. The data were analyzed using Applied Biosystems software “Sequence Detection Systems” Version 2.4.1. A manual threshold of 0.2 was used, and automatic baseline was applied. Data from outlier wells were excluded from the analysis. Δ*CT* (cycle threshold) values were calculated based on the mean *CT* values of the target genes and mean *CT* values of the reference control gene B2M, using Equation 4,

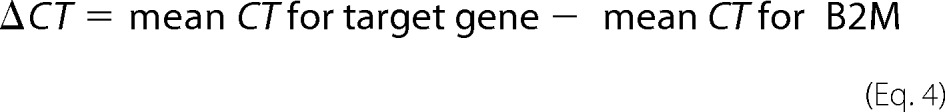


Relative gene expression levels were calculated using ΔΔ*CT* analysis and the normal lung as the calibrator sample as shown in Equation 5.

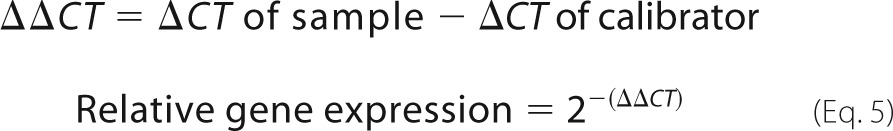


## Results

### 

#### 

##### Generation of Bispecific Abs for Receptor Quantitation and in Vitro Characterization

**FIGURE 1.**
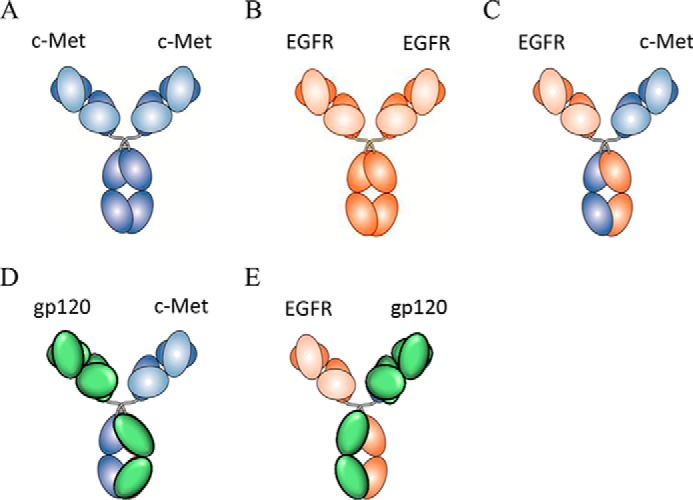
**Schematic of antibody molecules for receptor quantitation and target engagement studies.** *A,* α-c-MET bivalent parental mAb; *B,* α-EGFR bivalent parental Ab; *C,* α-EGFR × α-c-MET BsAb (JNJ-61186372); *D,* α-c-MET monovalent BsAb; and *E,* α-EGFR monovalent BsAb. The domains were colored as follows: *blue,* α-c-MET; *orange,* α-EGFR; *green,* α-gp120.

In this work, we sought to generate optimal reagents for quantifying EGFR and c-MET cell-surface expression and evaluating the target engagement of a BsAb (JNJ-61186372) targeting these two receptors. Because the stoichiometry of Ab binding cannot be predicted and because the impact of valency and avidity on the accuracy of receptor quantitation measurements have been well documented (38, 39), we chose to use a monovalent Ab system for quantitation. Additionally, understanding how a BsAb engages both its cell-surface targets can be challenging. Here, we harnessed the utility of cFAE technology for studies of both receptor quantitation and target engagement. We have taken high affinity anti-c-MET and anti-EGFR Abs and used cFAE to create BsAbs of >95% purity with >95% yield incorporating the following Fab arm combinations: one α-EGFR and one α-c-MET Fab arm (JNJ-61186372), one α-EGFR and one α-gp120 (HIV) Fab arm, and one α-c-MET Fab arm and one α-gp120 (HIV) Fab arm (Fig. 1). The α-gp120 (HIV) Fab arm is considered inert because none of the cells express the viral protein. Parental bivalent EGFR and c-MET Abs and their corresponding monovalent BsAbs with “inert” arms were conjugated to R-PE and used for receptor quantitation studies. The unlabeled Abs were used for phosphorylation and comparative cell binding assays.

Inert1 is an inert binding arm used to make monovalent BsAbs. These mAbs do not recognize targets that are relevant *in vitro*. Antibodies that lacked binding specificity to EGFR (inert1 × c-MET, c-MET parental mAb) did not bind to the EGFR-ECD. Because JNJ-61186372 was attached to the surfaces and the EGFR-ECD was monomeric, the *K_D_* values of the single arm EGFR binders (JNJ-61186372, and inert1 × EGFR) and the bivalent parental EGFR mAbs were similar. Hence in this format, the binding was reflective of single arm binding. Because the c-MET ECD-Fc was attached to the surface, the binding of JNJ-61186372 and inert1 × c-MET was reflective of single arm binding. The *K_D_* values of the c-MET arm in JNJ-61186372 and inert2 × c-MET were comparable.

**FIGURE 2.**
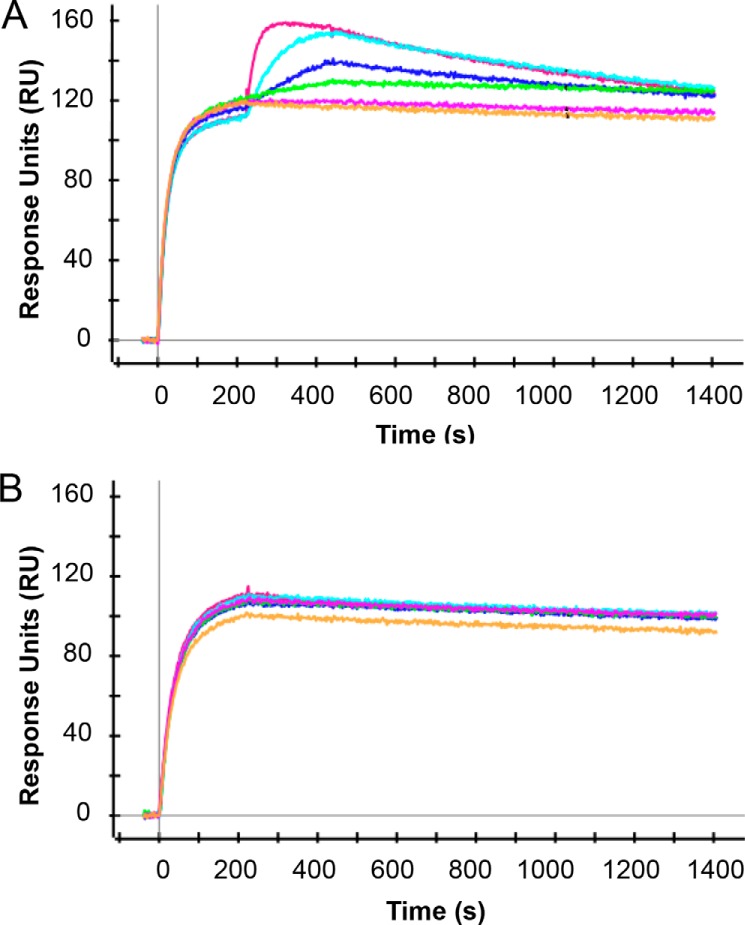
**JNJ-61186372 binds simultaneously to both c-MET and EGFR monomer.** c-MET-Fc-His was captured onto a HTG sensor chip. JNJ-61186372 (*A*) or inert arm × c-MET (*B*) was co-injected with serially diluted EGFR monomer over the captured c-MET-Fc-His molecule. The sensorgrams are shown per EGFR concentration in *red* (100 nm), *aqua blue* (25 nm), *blue* (6.25 nm), *green* (1.56 nm), *pink* (0.39 nm), and *orange* (0 nm).

JNJ-61186372 was able to bind to the EGFR after being bound to the c-MET receptor with an affinity of 1.4 ± 0.3 nm for the EGFR monomer (Fig. 2). This affinity is similar to that of the BsAb binding to EGFR in the absence of binding arm to c-MET (inert1 × EGFR binding to EGFR-ECD has a *K_D_* ∼1.4 ± 0.1 nm; EGFR parental mAb had *K_D_* ∼1.6 ± 0.2 nm). B21M × c-MET (a BsAb with c-MET binding arm but lacking the EGFR binding arm) did not bind to the EGFR monomer after being bound to the c-MET receptor. However, B21M × c-MET had a *K_D_* value for binding to c-MET ECD of 0.071 ± 0.02 nm, which was similar to that of JNJ-61186372 (*K_D_* ∼0.04 ± 0.01 nm) and parental c-MET mAb (*K_D_* <0.02 ± 0.002 nm).

##### Unimolar Labeling with (R)-Phycoerythrin

**FIGURE 3.**
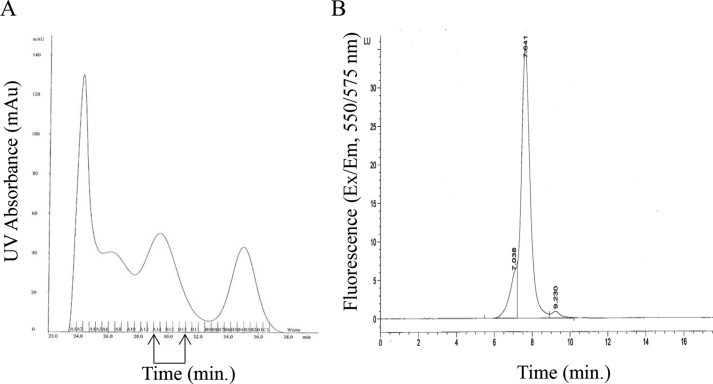
**Purification strategy of unimolar PE-labeled mAbs.** Example shown corresponds to c-MET × gp120 BsAb. *A,* SEC UV (280 nm) trace of PE-conjugated mAbs showing pooled fractions containing unimolar labeled material. *B,* results of analytical SEC showing ∼95% purity of unimolar PE-lableled mAb from pooled fractions. Free PE component is <10%.

The Abs used in this study were conjugated to phycoerythrin (R-PE) using heterobifunctional chemistry (see “Experimental Procedures”). Analytical HPLC of unimolar purified conjugates showed purities of ≥95% (Fig. 3). Commercially available Abs conjugated to a fluorochrome can be heterogeneous due to variable labeling and purity. Depending on the choice of fluorochrome and method of conjugation, the number of fluorochrome molecules conjugated to each Ab molecule can vary, and an F/P molar ratio of >1 introduces a degree of error into receptor quantitation calculations. In addition to its large size (240 kDa), which facilitates purification of unimolar labeled conjugates, the spectral characteristics of R-PE, which include a high quantum efficiency, high stain index, large Stokes shifts, and resistance to quenching, make it an ideal candidate for quantitative studies (38, 42). The use of R-PE conjugated in a 1:1 molar ratio to an Ab significantly improves the accuracy of QFCM methods (43). The percent recovery of this highly homogeneous purified material was ∼10%.

##### EGFR and c-MET Receptor Quantitation

**FIGURE 4.**
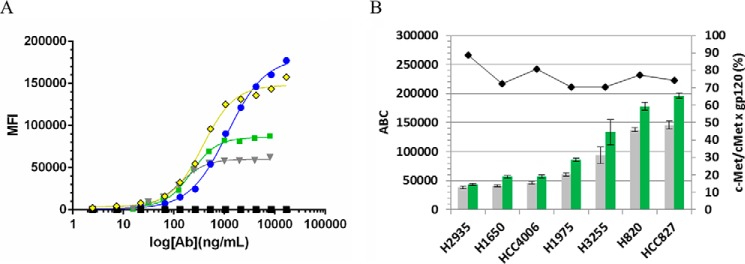
**Impact of valency on receptor quantitation.** *A,* exemplary binding curve of titrated PE-c-MET parental mAb (*gray* ▾), PE-c-MET × gp120 BsAb (*green* ■), PE-EGFR × gp120 BsAb (*blue* ●), PE-EGFR parental mAb (*yellow* ♦), and PE-IgG1 control (*black* ■) in HCC827 cells is shown. 4PL curve-fitting of log_10_ of Ab concentration *versus* MFI (geometric mean fluorescence intensity of PE) is shown. *B, left y* axis shows the specific ABC values ± S.E. using PE-c-MET parental mAb (*gray bar*) and PE-c-MET × gp120 BsAb (*green bar*) as determined for H2935, H1650, HCC4006, H1975, H3255, HCC827, and H820. *Right y* axis shows the ratio of ABC c-MET/ABC c-MET × gp120 expressed as a percentage.

We determined EGFR and c-MET expression levels on a panel of 11 tumor cell lines using QFCM. The determination of ABC was derived from a standard curve established by QuantiBRITE^TM^ PE beads. ABC values were determined for c-MET (Table 2) and EGFR (Table 3) using PE-labeled monovalent or bivalent Abs. As part of our initial investigation on the impact of valency on ABC value, we performed quantitative comparisons of monovalent *versus* bivalent EGFR and c-MET IgGs. Results showed that, at saturation, the monovalent Ab always gave a higher MFI value, and hence a greater ABC value (Fig. 4*A*).

**TABLE 2:** **ABC values for c-MET** Quantitation of c-MET levels using PE-labeled monovalent or bivalent Abs is shown. ABC values were derived from the geometric MFI of 5000 live events. ND means not determined.

PE-c-MET × gp120				PE-c-MET		
Cell line	Mean	S.E.	*n*	Mean	S.E.	*n*
H1975	8.61 × 10^4^	2.85 × 10^3^	6	6.06 × 10^4^	2.51 × 10^3^	6
HCC827	1.96 × 10^5^	4.32 × 10^3^	6	1.46 × 10^5^	6.94 × 10^3^	6
H1650	5.68 × 10^4^	2.47 × 10^3^	6	4.11 × 10^4^	1.71 × 10^3^	6
H3255	1.34 × 10^5^	2.20 × 10^4^	5	9.39 × 10^4^	1.43 × 10^4^	6
H820	1.78 × 10^5^	6.67 × 10^3^	6	1.37 × 10^5^	3.38 × 10^3^	6
H2935	4.32 × 10^4^	1.54 × 10^3^	2	3.84 × 10^4^	2.28 × 10^3^	2
HCC4006	5.69 × 10^4^	2.69 × 10^3^	2	4.60 × 10^4^	2.19 × 10^3^	2
H1993	5.61 × 10^5^	3.11 × 10^3^	4	ND		
H292	6.12 × 10^4^	1.00 × 10^3^	4	ND		
SKMES-1	4.60 × 10^4^	9.44 × 10^2^	3	ND		
SNU-5	4.93 × 10^5^	4.10 × 10^3^	4	ND		

**TABLE 3:** **ABC values for EGFR** Quantitation of EGFR levels using PE-labeled monovalent or bivalent Abs is shown. ABC values were derived from the geometric MFI of 5000 live events.

PE-EGFR × gp120				PE-EGFR		
Cell line	Mean	S.E.	*n*	Mean	S.E.	*n*
H1975	6.32 × 10^4^	2.56 × 10^3^	3	5.84 × 10^4^	2.01 × 10^3^	3
HCC827	4.01 × 10^5^	1.27 × 10^4^	2	3.48 × 10^5^	1.35 × 10^4^	4
H1650	1.21 × 10^5^	1741*^a^*	1	9.03 × 10^4^	8.45 × 10^3^	3
H3255	8.23 × 10^5^	15,039*^a^*	1	7.39 × 10^5^	3.19 × 10^4^	4
H820	1.11 × 10^5^	1.41 × 10^3^	2	9.25 × 10^4^	5.60 × 10^3^	2
H2935	1.34 × 10^5^	15,428*^a^*	1	8.24 × 10^4^	4380*^a^*	1
HCC4006	9.09 × 10^4^	7852*^a^*	1	5.05 × 10^4^	1850*^a^*	1
H1993	3.25 × 10^5^	7.99 × 10^3^	4	ND		
H292	3.57 × 10^5^	8.27 × 10^3^	2	ND		
SKMES-1	1.67 × 10^5^	7.72 × 10^3^	2	ND		
SNU-5	1.11 × 10^5^	6.96 × 10^3^	3	ND		

*^a^* S.E. for *n* = 1 was derived from nonlinear regression analysis of antibody concentration *versus* MFI. ND means not determined.

Although saturation was achieved for R-PE-c-MET Abs on all NSCLC cell lines, the R-PE-labeled EGFR Abs could not achieve saturation in all instances. When saturation could not be achieved, the maximum fluorescence intensity predicted by regression analysis of the logarithm of antibody concentration *versus* fluorescence was used for ABC determination. Although the bulky PE adduct could impact the binding of the EGFR Ab due to epitope location, cell line-specific receptor/co-receptor interactions, or some other type of steric hindrance, these labeled Abs had similar maximal MFI values as that of the unlabeled Abs detected by labeled secondary mAbs.

For a panel of seven NSCLC lines (H2935, H1650, HCC4006, H1975, H3255, HCC827, and H820) where direct comparisons were performed, the average Ab binding capacity of the bivalent c-MET Ab was 76.3 ± 6.2% of the Ab binding capacity of the monovalent c-MET (Fig. 4*B*). The difference in ABC values between the monovalent and bivalent parental Abs reflects the stoichiometry or avidity of Ab binding. Our preliminary results agreed with earlier studies by Davis *et al.* (38) who compared Ab and Fab binding to CD4 antigen on T cells or latex beads and found that the valency of binding was independent of antigen density. However, avidity can result in occlusion of receptor binding that prevents maximal binding of Abs on the cell surface. Likewise, we determined that the levels of monovalent Ab binding levels were higher than that of bivalent Ab binding. To most accurately phenotype the NSCLC lines for EGFR and c-MET, we assigned values for each receptor based on the monovalent Ab, which theoretically provides the most reliable estimate of the number of epitopes per cell.

##### Cell Culture and Time-dependent Changes in Receptor Expression

The impact of cell culture conditions, confluence, and passage number on expression levels has been previously described (39, 44). In this study, we have rigorously standardized cell culture procedures to minimize these sources of variability (see “Experimental Procedures”). The cell culture conditions used for the determination of receptor density, mRNA expression levels, and phosphorylation inhibition assays were identical. However, given the transformed nature of the NSCLC cell lines, some fluctuation in expression over time must be expected. The reproducibility of the EGFR and c-MET receptor ABC values varied by cell type and are listed in Tables 2 and 3.

##### Correlation of EGFR and c-MET mRNA Gene Expression to Receptor Density

**FIGURE 5.**
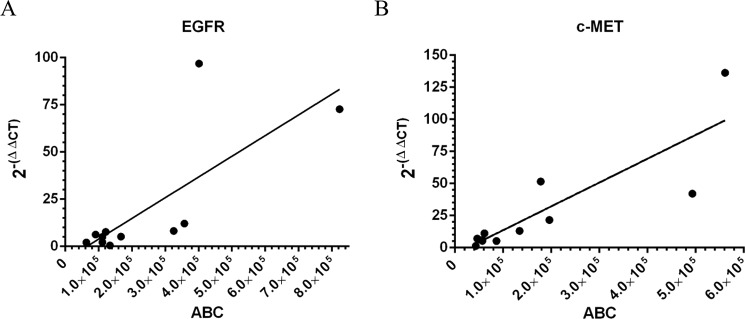
**Correlation of relative gene expression (2^−(ΔΔ^*^CT^*) to receptor quantitation.** *A,* mRNA levels for EGFR plotted *versus* EGFR ABC. Pearson correlation method results are *p* value = 0.0075 and *r*^2^ = 0.5663. *B,* mRNA levels for c-MET plotted *versus* c-MET ABC. Pearson correlation method results are *p* value = 0.0007 and *r*^2^ = 0.7368.

We performed expression profiling analysis of EGFR and c-MET for 11 NSCLC cell lines, and we normalized the values to the corresponding values measured in primary human lung fibroblasts (Table 4). Cell lines with amplified c-MET genes exhibited significantly higher mRNA levels than those with WT gene levels of c-MET (Tables 1 and 4). Normalized c-MET mRNA levels for H820, SNU-5, and H1993 were 52-, 42-, and 136-fold, respectively, whereas the WT cell lines ranged from 1- to 22-fold. Cell lines with the largest EGFR copy number, H3255 and HCC827 (Table 1), had the highest mRNA levels, 73- and 97-fold, respectively. However, not all EGFR-amplified cell lines showed EGFR mRNA levels higher than those with WT EGFR. WT EGFR mRNA levels ranged from 2- to 8-fold. Gene expression levels for both EGFR (Fig. 5*A*) and c-MET (Fig. 5*B*) did correlate in a statistically significant manner to receptor density ABC values. Pearson correlation coefficient for the c-MET gene-receptor density plot resulted in a *p* value of 0.0007 and an *r*^2^ of 0.7368. Pearson correlation coefficient for the EGFR gene-receptor density plot gave a *p* value of 0.0075 and *r*^2^ of 0.5663.

**TABLE 4:** **Relative mRNA levels of EGFR and c-MET**

	2^−(ΔΔ*CT*)^*^a^*	
Cell line	EGFR	c-MET
H1993	8.2	136.3
A549	3.0	7.8
H292	12.1	11.2
*Normal Lung*	*1.0*	*1.0*
SKMES-1	5.2	7.1
SNU-5	2.2	42.0
H3255	72.7	13.1
H1975	2.1	5.2
HCC4006	6.3	5.3
H1650	7.7	6.1
HCC827	96.9	21.6
H820	5.0	51.5
HCC2935	0.5	1.3

*^a^* See under “Experimental Procedures” for 2^−(ΔΔ*CT*)^ calculations.

##### Correlation of JNJ-61186372-mediated Inhibition of Receptor Phosphorylation to Receptor Density

**FIGURE 6.**
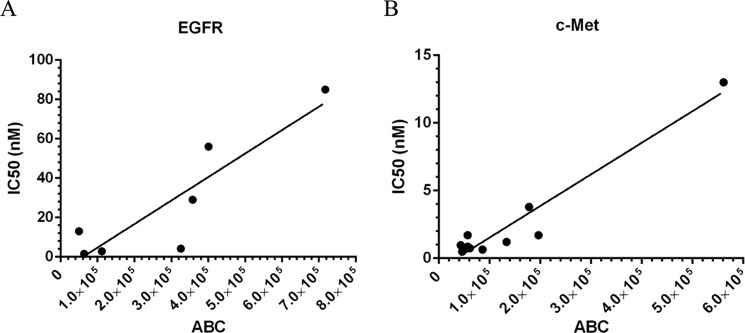
**Correlation of inhibition of receptor phosphorylation to receptor quantitation.** *A,* IC_50_ values for JNJ-61186372-mediated inhibition of EGFR phosphorylation plotted *versus* EGFR ABC. Pearson correlation method results are *p* = 0.0074 and *r*^2^ = 0.7903. *B,* IC_50_ values for JNJ-61186372-mediated inhibition of c-MET phosphorylation plotted *versus* c-MET ABC. Pearson correlation method results are *p* < 0.0001 and *r*^2^ = 0.9331.

**FIGURE 7.**
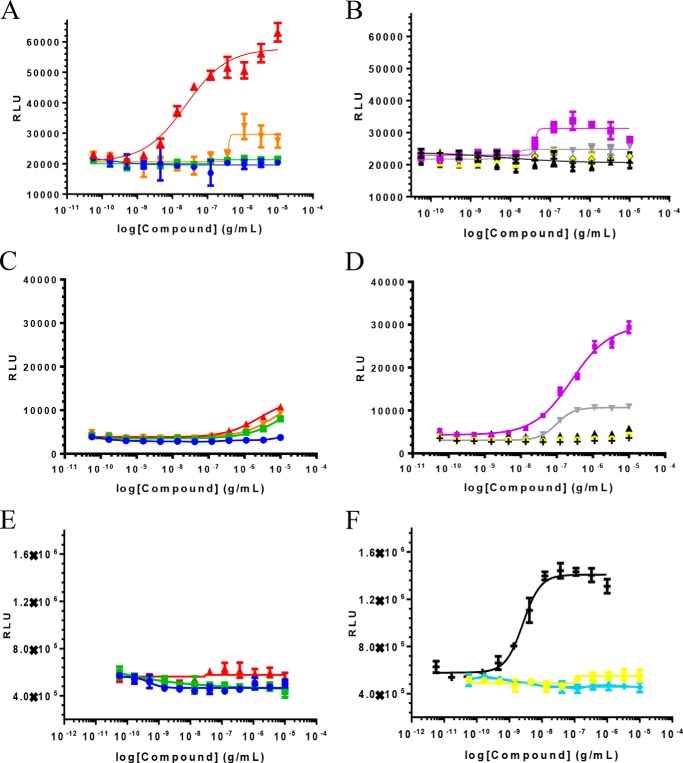
**Effect of JNJ-61186372 on the homo- and heterodimerization of EGFR and c-MET in U2OS cells.** Increase in EFC-mediated β-gal activity in EGFR/c-MET heterodimer expressing cells following incubation with EGFR × gp120 BsAb (*blue* ●), gp120 × c-MET BsAb (*green* ■), gp120 × c-MET + EGFR × gp120 (*orange* ▾), JNJ-61186372 (EGFR × c-MET) BsAb (*red* ▴) (*A*) and c-MET mAb (*gray* ▾), EGFR Ab (*yellow* ♦), HGF (*purple* ■), gp120 mAb (*black* ▴) and EGF (+) (*B*) is shown. Increase in EFC-mediated β-gal activity in c-MET homodimer expressing cells following incubation with EGFR × B21M BsAb (*blue* ●), B21M × c-MET BsAb (*green* ■), B21M × c-MET BsAb + EGFR × B2M BsAb (*orange* ▾), JNJ-61186372 (EGFR × c-MET) BsAb (*red* ▴) (*C*) and c-MET mAb (*gray* ▾), EGFR mAb (*yellow* ●), HGF (*purple* ■), B21M mAb (*black* ▴), and EGF (+) (*D*) is shown. Increase in EFC-mediated β-gal activity in EGFR homodimer expressing cells following incubation with EGFR × gp120 BsAb (*blue* ●), gp120 × c-MET BsAb (*green* ■), JNJ-61186372 (EGFR × c-MET) BsAb (*red* ▴) (*E*) and Erbitux (*blue* ♦), EGFR Ab (*yellow* ●), and EGF (**+**) (*F*) is shown. *RLU*, relative light units.

**FIGURE 8.**
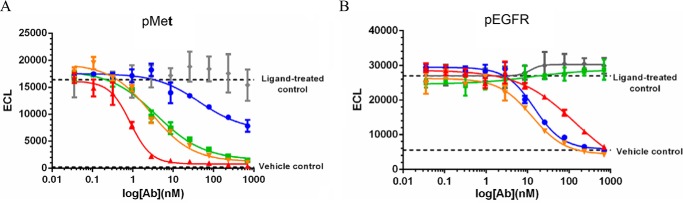
**Inhibition of ligand-induced receptor phosphorylation in H292 cells.** The ratio of EGFR to c-MET ABC values in H292 as measured by QFCM was 5.72-fold. 4PL indicates curve-fitting of log_10_ of Ab concentration *versus* ECL. Inhibition of c-MET phosphorylation relative to an HGF-treated control by EGFR × gp120 BsAb (*blu*e ●), gp120 × c-MET BsAb (*green* ■), gp120 × c-MET BsAb + EGFR x gp120 BsAb (*orange* ▾), and JNJ-61186372 (EGFR × c-MET) BsAb (*red* ▴), or gp120 mAb (*gray* ♦) is shown (*A*). Inhibition of EGFR phosphorylation relative to an EGF-treated control by EGFR × gp120 BsAb (*blu*e ●), gp120 × c-MET BsAb (*green* ■), gp120 × c-MET BsAb + EGFR × gp120 BsAb (*orange* ▾), and JNJ-61186372 (EGFR × c-MET) BsAb (*red* ▴), or gp120 mAb (*gray* ♦) is shown (*B*).

We determined IC_50_ values for the ability of JNJ-61186372 to block ligand-induced receptor phosphorylation of EGFR and c-MET on the panel of cell lines (Table 5). Representative dose responses are shown in Figs. 6–8. There was a correlation between normalized EGFR ABC values and the IC_50_ values of JNJ-61186372 against EGFR phosphorylation with a Pearson correlation coefficient *p* value of 0.0074 and an *r*^2^ value of 0.7903 (Fig. 6*A*). There was also a correlation between the normalized c-MET ABC and the IC_50_ values of JNJ-61186372 against c-MET phosphorylation with a Pearson correlation coefficient *p* value of <0.0001 and an *r*^2^ value of 0.9331 (Fig. 6*B*). In other words, the potency of inhibition of receptor phosphorylation by JNJ-61186372 was proportional to the receptor density for both EGFR and c-MET.

**TABLE 5:** **Inhibition of receptor phosphorylation** IC_50_ (values in nm) are calculated using GraphPad Prism 6 software. NF indicates no fit is used when either Prism does not return a value (*e.g.* “ambiguous”) or the fit is poor (95% confidence interval range is >4-fold). ND = not determined.

Molecule and receptor	H292			SNU-5			H1993		
	IC_50_	S.E.	*n*	IC_50_	S.E.	*n*	IC_50_	S.E.	*n*
**JNJ-61186372**									
Phosphorylated EGFR	29	ND	1	15	2.8	2	3.8	0.22	3
Phosphorylated c-MET	0.86	0.067	3	NF	ND	2	7.8	2.6	3

**EGFR × gp120**									
Phosphorylated EGFR	13	1.1	4	NF	ND	2	10	0.15	2
Phosphorylated c-MET	NF	ND	2	NF	ND	2	NF	ND	2

**c-MET × gp120**
Phosphorylated EGFR	NF	ND	4	NF	ND	2	NF	ND	2
Phosphorylated c-MET	4.1	0.15	2	NF	ND	2	7.8	ND	1

**EGFR × gp120 + c-MET × gp120**									
Phosphorylated EGFR	13	ND	1	NF	ND	2	9.2	0.21	2
Phosphorylated c-MET	2.8	ND	1	NF	ND	2	12	ND	1

##### JNJ-61186372 Heterodimerizes EGFR and c-MET

U-2 OS cells were stably transfected with EGFR and c-MET receptors genetically fused to complementary fragments of β-gal and optimized for expression. EFC assays were carried out by incubating the cells with serially diluted concentrations of either c-MET monovalent Ab, c-MET bivalent Ab, EGFR monovalent Ab, EGFR bivalent Ab, EGFR monovalent Ab + c-MET monovalent Ab, EGF, HGF, or bispecific JNJ-61186372. The samples were assessed for their ability to heterodimerize EGFR and c-MET as measured by β-gal activity. Treatment of the stably transfected U-2 OS cells with JNJ-61186372 elicited a significant dose-dependent increase in chemiluminescence (Fig. 7*A*) with a half-maximal effective concentration (EC_50_ of 142 ± 19.6 pm). The signal-to-noise (S/N) ratio for JNJ-61186372 in this assay was ∼2.8-fold. The combination of the single arm binding BsAbs had lower responses. Interestingly, HGF may also heterodimerize EGFR and c-MET but, at a S/N of ∼1.4, to a much lesser extent than JNJ-61186372 (Fig. 7*B*).

EGFR and c-MET homodimerization assays were also developed to validate the PathHunter® technology. A dose-dependent increase in β-gal activity was seen in response to EGF binding (EC_50_ of 10.1 ng/ml or 1.62 nm), with a S/N of ∼2.4. However, no EGFR homodimerization was evident upon incubation with any of the c-MET, EGFR Abs, or JNJ-61186372 (Fig. 7, *E* and *F*). No c-MET homodimerization was detected upon incubation with EGF and EGFR only Abs or BsAbs (Fig. 7, *C* and *D*). There was high level of c-MET dimerization upon addition of HGF (EC_50_ of 0.257 μg/ml or 3.21 nm), lower levels with bivalent c-MET mAb, but even lower levels of homodimerization upon incubation with JNJ-61186372 and monovalent c-MET BsAbs (gp120 × c-MET and the combination of gp120 × c-MET BsAb + EGFR × gp120 BsAb).

##### Avidity Effect of BsAbs in Inhibition of Receptor Phosphorylation

We also evaluated the potency with which JNJ-61186372 blocks ligand-induced receptor phosphorylation relative to c-MET × gp120 BsAb, EGFR × gp120 BsAb, or combinations of monovalent EGFR and c-MET BsAbs, in cell lines with different ratios of EGFR to c-MET expression. The ABC values for EGFR and c-MET in H292, H1993, and SNU-5 are reported in Tables 2 and 3. The Abs used in these comparisons were not PE-labeled.

Results showed that in H292 where the EGFR/c-MET ABC ratio was measured at ∼5.8 (Tables 2 and 3), the IC_50_ value of JNJ-61186372 in blocking c-MET receptor phosphorylation was 0.86 ± 0.067 nm, ∼5-fold lower than the IC_50_ value of c-MET × gp120 BsAb, which was 4.1 ± 0.15 nm (Fig. 8*A*). There was no inhibitory effect by the gp120 mAb and minimal inhibitory effect of EGFR × gp120 BsAb on c-MET phosphorylation. The small inhibitory effect of EGFR × gp120 BsAb on c-MET phosphorylation may be due to cross-talk between the two receptors. EGFR signaling has been shown to enhance HGF-induced c-MET phosphorylation levels in lung cancer cell lines (21). Additionally, the combination of EGFR × gp120 BsAb + c-MET × gp120 BsAb exhibited a limited increase in potency in blocking c-MET receptor phosphorylation relative to the c-MET × gp120 BsAb alone. The IC_50_ value of the EGFR × gp120 BsAb + c-MET × gp120 BsAb was 2.8 nm.

Interestingly, there was no increase in the potency of JNJ-61186372 in H292 in blocking EGF receptor phosphorylation relative to the EGFR × gp120 Ab BsAb alone (Fig. 8*B*). JNJ-61186372 had an IC_50_ value of 29 nm, whereas the EGFR × gp120 BsAb had an IC_50_ value of 13 ± 1.1 nm. There was no difference in potency between the EGFR × gp120 BsAb alone and the combination of EGFR × gp120 + c-MET × gp120, which also had an IC_50_ value of 13 nm. There was no inhibitory effect on EGFR phosphorylation for gp120 mAb or gp120 × c-MET BsAb. JNJ-61186372 therefore shows an increase in potency for c-MET phosphorylation inhibition and no increase in potency for EGFR phosphorylation inhibition in H292 where the EGFR receptor density is greater than the c-MET receptor density.

**FIGURE 9.**
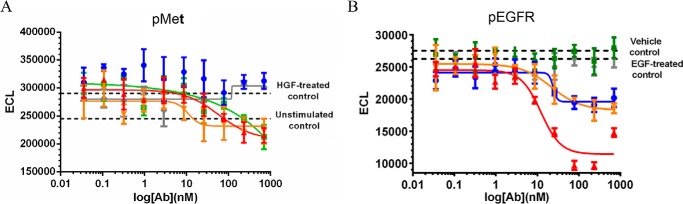
**Inhibition of ligand-induced receptor phosphorylation in SNU-5 cells.** The ratio of EGFR to c-MET ABC in SNU-5 as measured by QFCM was 0.2-fold. 4PL indicates curve-fitting of log_10_ of Ab concentration *versus* ECL. Inhibition of c-MET phosphorylation relative to an HGF-treated control by EGFR × gp120 BsAb (*blu*e ●), gp120 × c-MET BsAb (*green* ■), gp120 × c-MET BsAb + EGFR × gp120 BsAb (*orange* ▾), and JNJ-61186372 (EGFR × c-MET) BsAb (*red* ▴), or gp120 mAb (*gray* ♦) is shown (*A*). Inhibition of EGFR phosphorylation relative to an EGF-treated control by EGFR × gp120 BsAb (*blu*e ●), gp120 × c-MET BsAb (*green* ■), gp120 × c-MET BsAb + EGFR × gp120 BsAb (*orange* ▾), and JNJ-61186372 (EGFR × c-MET) BsAb (*red* ▴), or gp120 mAb (*gray* ♦) is shown (*B*).

We also compared these results with cell lines that have an inverse relationship of ABC values of EGFR and c-MET receptor densities. In SNU-5 cells, where the EGFR/c-MET-specific ABC ratio was measured at ∼0.2 (Tables 2 and Table 3), the IC_50_ value of JNJ-61186372 in blocking EGFR receptor phosphorylation was 15 ± 2.8 nm, whereas no IC_50_ value could be derived from the EGFR × gp120 titration (Fig. 9*B*). The combination of EGFR × gp120 + c-MET × gp120 did have a slight inhibitory effect on EGFR phosphorylation, although an IC_50_ value could not be derived. Because of the high levels of c-MET present, there was a high background of c-MET phosphorylation activity (Fig. 9*A*). In fact, no IC_50_ values could be generated for blocking c-MET receptor phosphorylation in SNU-5.

**FIGURE 10.**
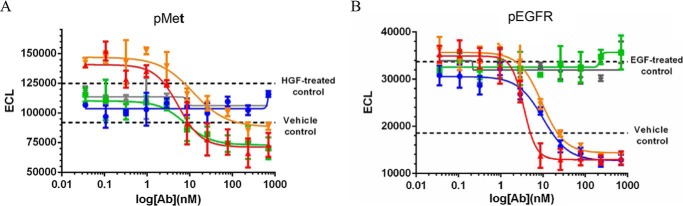
**Inhibition of ligand-induced receptor phosphorylation in H1993 cells.** The ratio of EGFR to c-MET ABC in H1993 as measured by QFCM was 0.6-fold. 4PL indicates curve-fitting of log_10_ of Ab concentration *versus* ECL. Inhibition of c-MET phosphorylation relative to an HGF-treated control by EGFR × gp120 BsAb (*blu*e ●), gp120 × c-MET BsAb (*green* ■), gp120 × c-MET BsAb + EGFR × gp120 BsAb (*orange* ▾), and JNJ-61186372 (EGFR × c-MET) BsAb (*red* ▴), or gp120 mAb (*gray* ♦) is shown (*A*). Inhibition of EGFR phosphorylation relative to an EGF-treated control by EGFR × gp120 BsAb (*blu*e ●), gp120 × c-MET BsAb (*green* ■), gp120 × c-MET BsAb + EGFR × gp120 BsAb (*orange* ▾), and JNJ-61186372 (EGFR × c-MET) BsAb (*red* ▴), or gp120 mAb (*gray* ♦) is shown (*B*).

We performed the same experiments in H1993 cells where the EGFR density is less than that of c-MET, as in SNU-5 cells. In H1993 cells, the EGFR/c-MET ABC ratio was measured at ∼0.6 (Tables 2 and 3). The IC_50_ value of JNJ-61186372 in blocking EGFR phosphorylation was 3.8 ± 0.22 nm, which was 2.6-fold lower than the IC_50_ value of EGFR × gp120 alone, which was 10 ± 0.15 nm (Fig. 10*A*). There was no inhibitory effect of the c-MET × gp120 BsAb and gp120 mAb alone on EGFR phosphorylation. The IC_50_ value of the EGFR × gp120 BsAb + c-MET × gp120 BsAb combination was 9.2 ± 0.21 nm, slightly more potent at blocking EGFR phosphorylation than EGFR × gp120 BsAb alone. The IC_50_ value of JNJ-61186372 at blocking c-MET receptor phosphorylation was 7.8 ± 2.6 nm, nearly identical to the IC_50_ value of c-MET × gp120 BsAb alone, which was 7.8 nm. The IC_50_ value of the EGFR × gp120 BsAb + c-MET × gp120 BsAb combination on blocking c-MET receptor phosphorylation was 12 nm (Fig. 10*B*). There was no inhibitory effect of the EGFR × gp120 BsAb alone or gp120 mAb on c-MET phosphorylation. In summary, JNJ-61186372 shows an increase in potency for EGFR phosphorylation inhibition and no increase in potency for c-MET phosphorylation inhibition in SNU-5 and H1993 cells where the c-MET receptor density is greater than the EGFR density. In conclusion, the increase in BsAb phosphorylation inhibition levels follow the relative ratios of the target receptors on a cell surface.

##### JNJ-61186372 Binding to Cells Is Guided by the More Highly Expressed of Its Two Targeted Receptors

**FIGURE 11.**
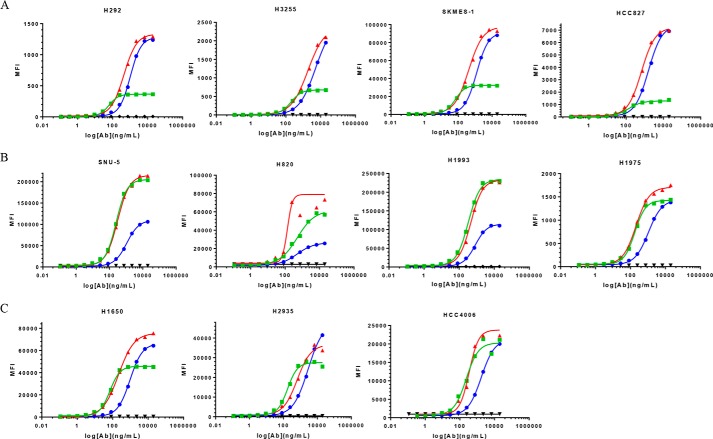
**Cell binding comparisons of JNJ-61186372 (EGFR × c-MET) BsAb, c-MET × gp120 BsAb, and EGFR × gp120 BsAb.** 4PL indicates curve-fitting of log of Ab concentration *versus* MFI. *A,* cell lines for which JNJ-61186372 approximates EGFR × gp120. *B,* cell lines for which JNJ-61186372 approximates c-MET × gp120 BsAb. *C,* cell lines for which JNJ-61186372 binding shows a less discernible pattern. EGFR × gp120 BsAb (*blu*e ●), c-MET × gp120 BsAb (*green* ■), JNJ-61186372 BsAb (*red* ▴), IgG1 isotype control (*gray* ♦).

In an attempt to understand how a BsAb engages its surface targets, we performed comparative binding flow cytometry titrations with JNJ-61186372, c-MET × gp120 BsAb, and the EGFR × gp120 BsAb against multiple cell lines. We used unlabeled primary Abs followed by PE-labeled secondary Ab detection. A qualitative examination of the binding curves revealed an interesting pattern. The JNJ-61186372 curves approximate the curve of the monovalent Ab corresponding to the more highly expressed receptor in a given cell line (Fig. 11, *A* and *B*). In Fig. 11*A*, JNJ-61186372 was more potent in binding than the monovalent EGFR × gp120 and c-MET × gp120 BsAbs. In Fig. 11*A*, the ABC EGFR/ABC c-MET ratios were 10.7 for H292; 6.1 for H3255 and 3.6 of SKMES-1; and 2.0 for HCC827. Thus, the cell binding profiles were more similar to binding to EGFR alone as exemplified by the binding profiles of EGFR × gp120 BsAbs as compared with that of c-MET × gp120. Conversely, in Fig. 11*B*, JNJ-61186372 was more potent or equipotent in binding than the monovalent EGFR × gp120 and c-MET × gp120 BsAbs. In Fig. 11*B*, the ABC EGFR/ABC c-MET ratios were 0.22 for SNU-5; 0.62 for H820 and 0.58 of H1993; and 0.72 for H1975. Thus, the cell binding profiles were more similar to binding to c-MET alone as exemplified by the binding profiles of c-MET × gp120 BsAbs as compared with that of EGFR × gp120. The greater the specific ABC difference between EGFR and c-MET, the more clearly this pattern is evident. There appears to be saturation of binding for JNJ-61186372 that is not much greater than that of the respective single arm BsAb. However, this pattern was less discernible for the H2935, H1650, and HCC4006 where the ABC EGFR/ABC c-MET ratios were 3.1 for H2935; 2.1 for H1650 and 1.6 for HCC4006 (Fig. 11*C*). For these cell lines, there were lower levels of c-MET ABC values as compared with cell lines that were EGFR-driven (H292, H3255, SKMES-1, and HCC827) or c-MET-driven (SNU-5, H820, H1993, and H1975).

## Discussion

BsAbs generated by cFAE (Fig. 1) are novel and convenient tools with which to carry out quantitation and comparative binding studies. The incorporation of a nonbinding arm targeting an extraneous antigen obviates the need to switch to an entirely different molecular format (*e.g.* Fab and scFv) to secure monovalency. This convenience removes potential biases that might occur in comparative studies if disparate spatial configurations impact how the molecules interact with surface targets. cFAE-derived monovalent Abs were labeled with PE and purified to obtain a 1:1 F/P ratio (Fig. 3) for the purpose of quantitation. The latter removes the procedural requirement to account for multiple or heterogeneous labeling when interpreting fluorescence data. By keeping the architecture of the BsAbs, mAbs, and monovalent Abs consistent, we can minimize concerns about the binding and activity data due to changes in spatial geometry.

The SPR assay confirms that JNJ-61186372 can bind to c-MET and EGFR simultaneously (Fig. 2). The EFC assay results (Fig. 7) suggested that JNJ-61186372 could simultaneously engage (*i.e.* heterodimerize) EGFR and c-MET, which may already be in close proximity on the cell surface. Jo *et al.* (33) found that EGFR and c-MET co-immunoprecipitated in cancer cells and suggested that their association facilitates the HGF-independent phosphorylation of c-MET through TGFα/EGF-mediated EGFR activation.

A thorough investigation of binding stoichiometry for c-MET in a panel of NSCLC cell lines (Fig. 4) showed that cFAE-generated monovalent Abs gave consistently higher ABC values than the bivalent Abs. We propose that, as a result of their 1:1 receptor-binding stoichiometry, the ABC values generated using monovalent Abs more closely approximate the true number of cell-surface epitopes. Although not the primary focus of this study, it should be noted that cFAE provides a unique opportunity to investigate the stoichiometry of binding by allowing a direct comparison between two full-length IgGs, in bivalent and monovalent format.

We found that the receptor numbers were correlated to *in vitro* inhibition of EGFR and c-MET receptor phosphorylation by EGFR × c-MET bispecific JNJ-61186372 (Fig. 6). Because inhibition of cell-surface receptor phosphorylation is the immediate functional activity mediated by a neutralizing anti-EGFR or anti-c-MET Ab, a methodology that yields reasonably accurate target-receptor quantification should produce this correlation. We found no correlation between receptor density values and the inhibition of downstream signaling in the AKT and ERK pathways (data not shown). Given the complexity and cross-talk in the EGFR and c-MET signaling cascades, the lack of correlation between receptor levels and downstream signaling events is perhaps to be expected. We also found a statistically significant correlation between receptor number and quantitative PCR-determined gene expression (data not shown). However, we realize that intervening post-transcriptional and post-translational regulatory mechanisms may render the latter correlation fortuitous or biologically irrelevant.

The potency with which JNJ-61186372 blocks ligand-induced receptor phosphorylation is greater than that of the corresponding monovalent Ab directed against that receptor. This effect is most pronounced against the less highly expressed of the targeted EGFR/c-MET pair. We have shown this effect against both c-MET (Fig. 8) and EGFR (Figs. 9 and 10) in cell lines in which those receptors were quantitatively determined to be the less highly expressed (Tables 2 and 3). The effect was most pronounced in H292 (Fig. 8), in which EGFR expression is nearly 11-fold greater than that of c-MET. In SNU-5, the inability to block c-MET phosphorylation is likely due to ligand-independent constitutive phosphorylation that results in the high background that cannot result in higher response in the presence of ligand (Fig. 9). The intriguing and unexpected ligand-independent EGFR phosphorylation observed in SNU-5 is perhaps also the result of cross-talk between EGFR and the amplified c-MET (21). We conjectured that the enhanced potency of bispecific JNJ-61186372 relative to monovalent EGFR × gp120 in SNU-5 might be due to receptor degradation. However, neither downstream signaling (pERK and pAKT) nor proliferation was affected by JNJ-61186372 (data not shown). Nonetheless, there was enhanced potency found in EGFR phosphorylation inhibition in H1993 cells that have the ABC EGFR/ABC c-MET ratios of 0.6 (Fig. 10). Thus, JNJ-61186372 enhancement in c-MET phosphorylation inhibition occurred in cell lines with higher EGFR and lower c-MET receptor density levels. But the JNJ-61186372 enhancement in EGFR phosphorylation inhibition occurred in cell lines with lower EGFR and higher c-MET receptor density levels.

**FIGURE 12.**
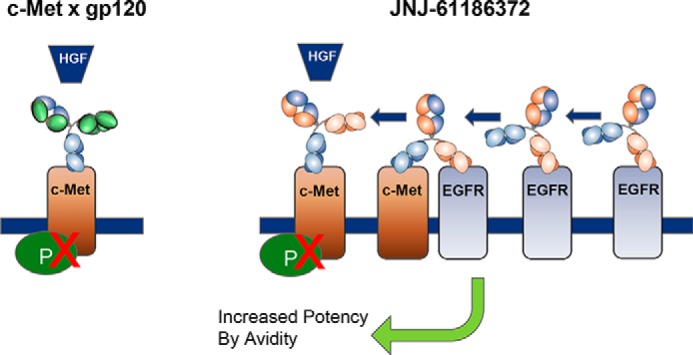
**Model for BsAb target engagement.** High EGFR to c-MET expression confers an increased potency to JNJ-61186372 BsAb over a monovalent c-MET BsAb in its ability to block HGF-mediated phosphorylation.

We hypothesize that the extent to which JNJ-61186372 interacts with target cells is determined initially by whichever of the two receptors, EGFR or c-MET, is more highly expressed. That initial interaction increases the local cell-surface concentration of the BsAb. Subsequently, it contacts the less highly expressed target, whereupon dual-receptor avidity results in the balanced, enhanced inhibition of the EGFR/c-MET signaling system that we have observed (Fig. 12). Moreover, JNJ-61186372 may sequester the EGFR and c-MET receptors into a nonsignaling heterodimeric complex that prevents their homodimerization, thus contributing to the increased *in vitro* potency of JNJ-61186372 relative to that of the monovalent EGFR or c-MET Abs.

These results are also consistent with those of Castoldi *et al.* (21) who demonstrated that an EGFR × c-MET BsAb could preclude the c-MET-driven tumor spreading that results from unbalanced EGFR inhibition in the presence of HGF. We corroborate their conclusions by demonstrating a distinct advantage of working with BsAbs, *i.e.* their intrinsic capacity to achieve balanced inhibition of interactive receptor systems such as EGFR and c-MET.

Affinity, target antigen density, and Ab binding are highly interdependent (2), *i.e.* the binding of a low affinity Ab will be influenced by avidity to a much greater extent than the binding of a high affinity Ab. Malignant cells frequently express certain antigens at significantly higher levels than normal cells (9–11). Therefore, as long as the affinity of either arm is not too high, a bispecific Ab that binds to at least one overexpressed target on a cancer cell should, as a result of dual-receptor avidity, preferentially bind to tumor cells rather than normal cells. This argument suggests how target antigen density and affinity might be used to guide BsAb design. For example, if one of two targeted antigens is significantly overexpressed in tumor *versus* normal cells, it might be advantageous to have the corresponding arm of the BsAb be of relatively low affinity to minimize its binding healthy cells. Similarly, when both targeted antigens are overexpressed on the cancer cells, engineering lower affinities into both arms of the BsAb should minimize interactions with healthy cells. In both cases, dual-target avidity would drive preferential accumulation of the BsAb onto the target cells, thus securing both specificity and efficacy.

We confirmed that expression differences can be used to decipher the mechanisms of BsAb target engagement and target occupancy on the surface of cancer cells. The differences of receptor density levels can affect bispecific Ab efficacy. Thus panels of BsAbs, which can be readily generated by cFAE, should be screened using variable pools of parental mAbs to determine empirically the most effective target affinity combinations. The paradigm that cancer cells could be preferentially targeted via dual-target avidity traps with BsAbs of calibrated affinities could be tested *in vitro* and prototyped *in vivo* using xenograft models. Such characterizations can be applied to target cells for indications other than cancer.

## Author Contributions

S. W. J. and J. P. conducted the flow assays and coordinated the mRNA analyses and heterodimerization assays shown in Figs 4–7 and 11; K. B. and E. R. L. conducted the SPR binding assays shown in Fig. 2; R. S. and M. A. S. designed and isolated the PE labeling that was used in Fig. 2; B. S. B. and S. L. M. designed and conducted the phosphorylation inhibition assays shown in Figs. 8–10; M. L. C. conceived and coordinated the work in this paper. All authors reviewed the results, contributed to the writing, and approved the final version of the manuscript.

## Footnotes

2 The abbreviations used are:

NSCLC: non-small cell lung cancer
Ab: antibody
ABC: antibody binding capacity
B2M: β2 microglobulin
BsAb: bispecific antibody
cFAE: controlled Fab-arm exchange
HGF: hepatocyte growth factor
MFI: mean fluorescence intensity
4PL: four parameter logistic
EGFR: epidermal growth factor receptor
QFCM: quantitative flow cytometry
R-PE: (*R*)-phycoerythrin
SEC: size-exclusion chromatography
NEAA: nonessential amino acid
EFC: enzyme fragment complementation
SPR: surface plasmon resonance
F/P: fluorochrome to protein
CV: column volume
S/N: signal to noise ratio.
